# Functional and structural evaluation in the lungs of children with repaired congenital diaphragmatic hernia

**DOI:** 10.1186/s12887-021-02586-3

**Published:** 2021-03-11

**Authors:** June-Young Koh, Euiseok Jung, Hyun Woo Goo, Seong-Chul Kim, Dae Yeon Kim, Jung-Man Namgoong, Byong Sop Lee, Ki-Soo Kim, Ellen Ai-Rhan Kim

**Affiliations:** 1 Departments of Pediatrics, Asan Medical Center, University of Ulsan College of Medicine, Seoul, Republic of Korea; 2 Graduate School of Medical Science and Engineering, Korea Advanced Institute of Science and Technology (KAIST), Daejeon, Republic of Korea; 3 Department of Radiology and Research Institute of Radiology, Asan Medical Center, University of Ulsan College of Medicine, Seoul, Republic of Korea; 4 Departments of Pediatric Surgery, Asan Medical Center, University of Ulsan College of Medicine, Seoul, Republic of Korea

**Keywords:** Congenital diaphragmatic hernia, Pulmonary function tests, Computed tomography, Risk factors

## Abstract

**Background:**

To evaluate the long-term functional and structural pulmonary development in children with repaired congenital diaphragmatic hernia (CDH) and to identify the associated perinatal-neonatal risk factors.

**Methods:**

Children with repaired CDH through corrective surgery who were born at gestational age ≥ 35 weeks were included in this analysis. Those who were followed for at least 5 years were subjected to spirometry and chest computed tomography for evaluation of their functional and structural growth. Main bronchus diameters and lung volumes (total, left/right) were measured. According to total lung volume (TLV) relative to body surface area, children were grouped into TLV ≥ 50 group and TLV < 50 group and the associations with perinatal-neonatal factors were analyzed.

**Results:**

Of the 28 children (mean age, 6.2 ± 0.2 years) with left-sided CDH, 7 (25%) had abnormal pulmonary function, of whom 6 (87%) showed restrictive patterns. All pulmonary functions except FEF25–75% were worse than those in matched healthy control group. Worse pulmonary function was significantly associated with small head and abdominal circumferences at birth. The mean TLV was 1339.1 ± 363.9 mL and LLV/TLV was 47.9 ± 2.5 mL. Children with abnormal pulmonary function were more likely to have smaller lung volumes. In multivariate analysis, abdominal circumference at birth was significantly associated with abnormal lung volume.

**Conclusions:**

A quarter of children with repaired CDH showed abnormal pulmonary function. Small abdominal circumference at birth was associated with abnormal pulmonary function and lower TLV.

**Supplementary Information:**

The online version contains supplementary material available at 10.1186/s12887-021-02586-3.

## Background

Congenital diaphragmatic hernia (CDH) is a rare disease that affects approximately 1 in 2500 newborns [1, 2]. The herniation of abdominal organs into the thoracic cavity inhibit the growth of the bronchioles, alveoli, and pulmonary vessels, thereby resulting in pulmonary morbidities [3, 4]. As such, up to 30% of fetuses with CDH die before birth and 30% of infants expire due to respiratory failures accompanying persistent pulmonary hypertension.

With the recent advances in neonatal intensive care [5, 6], the survival rates of neonates with repaired CDH increased [7, 8] and the interest in compensatory growth and maturation of the hypoplastic lungs also increased. Several studies reported that children with repaired CDH have complex restrictive and obstructive pulmonary disorders especially in the small airway; moreover, such patients also show ventilation-perfusion mismatches [9–11]. Multiple studies have analyzed the growth of lung parenchyma in children with repaired CDH and reported that the disease-involved lungs show catch-up growth [12, 13], albeit to a lesser degree than the contralateral side with findings with such as air trapping and architectural distortion. However, no studies to date have investigated lung development in such children after the neonatal period or analyzed the associations between functional impairment and structural underdevelopment.

Herein, we describe the long-term pulmonary morbidities both in functional and structural aspects and report the associated perinatal-neonatal risk factors in children with repaired CDH.

## Methods

### Subjects

We identified neonates with CDH born between January 1, 1994 and December 31, 2012 at Asan Medical Center and underwent corrective surgery in the neonatal intensive care unit; among them, we included those whose follow-up was possible after 5 years in the analysis. Premature infants (delivered at gestational age < 35 weeks) or infants with congenital malformations, congenital heart disease, or chromosome abnormalities were excluded from the analysis.

This research was reviewed and approved by the institutional review board of Asan Medical Center (Seoul, Republic of Korea; 2021–0238). Informed consent was obtained from the patients’ parents.

### Clinical data of the perinatal-neonatal period

We collected radiographic images and perinatal-neonatal clinical data including sex, birth date, birth weight, gestational age at delivery, mode of delivery, 1 min/5 min Apgar scores, fetus hydrops, polyhydramnios, date of surgery from birth, duration of high-frequency ventilation therapy, treatment with nitric oxide, use of extracorporeal membrane oxygenator, oxygenation index, persistent pulmonary hypertension, head circumference, chest circumference, and abdominal circumference upon admission to NICU. Regarding CDH, we gathered information on the herniation site, size of the defect (expressed as the product of the maximum diameter and the maximum diameter perpendicular of the defect), and the number and type of herniated organs found during surgery.

### Pulmonary function test

Pulmonary function tests (PFT) were performed on an outpatient basis under the supervision of a skilled pediatric respiratory specialist. Forced vital capacity (FVC), forced expiratory volume in 1 s (FEV1), FEV1/FVC ratio (FEV1/FVC) and maximum mid-expiratory flow were measured using spirometry (Sensor type, Dry rolling type, Vmax® Encore PFT system, Viasys), and the respective values of variables were converted to percentages from normal prediction value according to the American Thoracic Society and European Respiratory Society guidelines [14] based on age, sex, height, and race. The obtained data were compared with those of healthy controls of similar age, sex, and race collected by the Department of Pulmonology at our center. Children with restrictive and obstructive pulmonary dysfunction were defined as those with FVC less than 80% of the predicted value and those with FEV1/FVC less than 85% of the predicted value, respectively.

### Chest computed tomography

Chest computed tomography (CT) was performed to evaluate the structural growth of the lungs. CT was performed using electrocardiogram-synchronized cardiac CT with a 128-slice dual-source scanner. The obtained images were reconstructed and read by skilled pediatric radiologists who measured the left and right main bronchus inner diameters and left and right lung volumes. The sum of the left and right lung volumes were defined as the total lung volume (TLV), and the total lung volume relative to the body surface area was calculated. Total lung volume (TLV) was compared to the expected mean TLV in sex- and age-matched healthy children. Children with TLV below the 50th percentile of healthy children were assigned to the TLV < 50 group, and those with TLV equal to or above the 50th percentile of healthy children were assigned to the TLV ≥ 50 group [15]. We defined the value of the left lung volume relative to total lung volume as LLV/TLV.

### Statistical analysis

IBM SPSS Statistics for Windows ver 21.0 (IBM Corp., Armonk, NY, USA) and GraphPad PRISM ver 6.01 (GraphPad Software, San Diego, CA, USA) was used for statistical analysis. All clinical indices were described using mean and standard deviation of the values. Student’s t-test was used to compare the results of pulmonary function tests between the healthy control group and children with repaired CDH. Chi-squared analysis and Student’s t-test were used to evaluate the correlation between perinatal-neonatal clinical information, pulmonary function tests after the growth, and computational tomography test. For risk factor analysis, multivariate analysis with backward elimination was performed.

## Results

### Neonatal characteristics

A total of 30 children with repaired CDH were included in the analysis (Supplementary Table 1). Of those, 28 (93%) had left diaphragmatic hernia. Of the two cases of right CDH, one case had a defect size of 48 cm^2^, and the small bowel, colon, spleen, stomach, and liver were herniated. The other case of right CDH had a defect size of 12 cm^2^, and the small bowel and colon were herniated. On average, surgery for hernia was performed at 4.1 ± 1.8 days after birth (Supplementary Table 2).

### Pulmonary function test findings in children with repaired CDH and healthy control group

Preschool children with repaired left CDH (*n* = 28) were selected for pulmonary function tests. The average age at which the pulmonary function test was carried out was 6.2 ± 0.2 years. The pulmonary indices of age-, sex-, and race-matched healthy control group were selected from a previous study carried out at our center. The pulmonary function indices of the healthy control group were similar to the normal predicted values. In contrast, the pulmonary function indices of the children with repaired left CDH were significantly lower than those of the control group (all *P-value* < 0.01) except for FEV1/FVC (*P-value* = 0.5) and FEF25_75% (*P-value* = 0.5) (Table 1).

**Table 1:** Pulmonary function test findings in preschool children with congenital diaphragmatic hernia and healthy control group^a^

	Case (*n* = 28)	Control (*n* = 38)	Mean difference	*P-value*
Male, n (%)	15 (53%)	19 (50%)	–	0.80
PFT age^b^, years	6.2 ± 0.2	6.2 ± 0.1	–	0.96
FVC, liter	1.2 ± 0.1	1.5 ± 0.04	0.3 ± 0.1	< 0.001
FVC%, %	91.2 ± 2.6	107.4 ± 1.6	16.2 ± 3.1	< 0.001
FEV_1_, liter	1.1 ± 0.05	1.4 ± 0.04	0.3 ± 0.1	< 0.001
FEV_1_%, %	90.7 ± 2.7	108.4 ± 1.9	17.8 ± 3.3	< 0.001
FEV_1_/FVC, %	91.1 ± 1.3	92.1 ± 0.9	1.1 ± 1.6	0.50
FEF_25_75%_, liter/second	1.5 ± 0.1	1.9 ± 0.1	0.4 ± 0.1	< 0.01
FEF_25_75%_%, %	98.8 ± 5.7	103.7 ± 4.8	5.0 ± 7.5	0.50

*FEF*_*25_75%*_ maximum mid-expiratory flow, *FEV*_*1*_ forced expiratory volume in 1 s, *FEV*_*1*_*/FVC* FEV_1_ to FVC ratio, *FVC* functional vital capacity

^a^Children with left-sided congenital diaphragmatic hernia only

^b^Age at which pulmonary function test was performed

### Comparison of pulmonary function test and neonatal variables

To analyze the risk factors associated with pulmonary function deterioration, clinical indices of the perinatal-neonatal period were compared between children with normal PFT (*n* = 21) and those with pulmonary dysfunctions including restrictive/obstructive pulmonary impairment (*n* = 7). Pulmonary dysfunction, restrictive pulmonary impairment, and obstructive pulmonary impairment were identified in 7 (25%), 6 (21%), and 1 (4%) children, respectively. Age, birth weight, and gestational age were not significantly different between the two groups. Compared with those with normal PFT, the group with abnormal PFT had smaller head circumference (*P-value* < 0.05), chest circumference (*P-value* < 0.001), and abdominal circumference (*P-value* < 0.05) at birth (Table 2). There were no significant differences in the size of the defect, the number and type of herniated organs, date of surgery, usage of high-frequency oscillatory ventilation, nitric oxide, and treatment of cardiopulmonary circulatory system. Children with abnormal PFT showed longer durations of hospitalization tendency than the normal group (30.7 vs. 21.5 days), albeit the difference was not statistically significant.

**Table 2:** Perinatal, surgical, and neonatal variables in children with normal or abnormal pulmonary function test results^a^

	Normal PFT (*n* = 21)	Abnormal PFT (*n* = 7)	*P-value*
PFT age^b^, years	6.2 ± 1.0	6.4 ± 0.9	0.5
Perinatal factors			
Male, n (%)	14 (67%)	2 (29%)	0.1
Birth weight, g	3024.9 ± 294.0	2775.9 ± 325.9	0.1
Gestational age, weeks	38.5 ± 1.1	38.3 ± 1.1	0.7
1 min APAGAR score	5.8 ± 1.5	6.2 ± 1.4	0.5
5 min APAGAR score	7.8 ± 1.1	7.9 ± 1.2	0.8
HC^c^, cm	34.9 ± 1.1	33.6 ± 1.1	< 0.05
CC^c^, cm	32.3 ± 1.5	30.3 ± 1.0	< 0.001
AC^c^, cm	29.3 ± 1.5	26.9 ± 1.0	< 0.05
HC/CC^c^	1.1 ± 0.1	1.1 ± 0.1	0.4
Polyhydramnios, n (%)	3 (14%)	1 (14%)	0.7
Surgical findings			
Herniated organs, n	3 ± 1.0	2.7 ± 1.1	0.6
Stomach, n (%)	11 (52%)	2 (29%)	0.3
Small bowel, n (%)	20 (95%)	7 (100%)	0.8
Colon, n (%)	17 (81%)	4 (57%)	0.2
Liver, n (%)	3 (15%)	1 (14%)	0.7
Spleen, n (%)	13 (62%)	5 (72%)	0.5
Kidney, n (%)	1 (5%)	0 (0%)	0.8
Defect size, cm^2^	13.0 ± 9.8	8.1 ± 6.5	0.2
Neonatal factors			
Days to surgery	4.0 ± 1.1	4.4 ± 1.9	0.6
HFOV, n (%)	8 (38%)	3 (43%)	0.5
iNO, n (%)	1 (5%)	1 (14%)	0.4
ECMO, n (%)	0 (0%)	1 (14%)	0.2
Max FiO_2_^d^	0.6 ± 0.2	0.4 ± 0.3	0.3
Max OI^e^	9.5 ± 14.0	12.8 ± 13.9	0.6
Mean OI^f^	3.5 ± 2.0	4.8 ± 4.3	0.4
Preop Mean OI^g^	4.9 ± 4.5	8.0 ± 7.6	0.3
Duration of ventilation, days	7.8 ± 4.7	12.6 ± 9.0	0.2
Hospital day	21.5 ± 9.3	30.7 ± 18.0	0.09
PPHN, n (%)	10 (48%)	3 (43%)	0.6
Pneumothorax, n (%)	1 (5%)	0 (0%)	0.7
Multivariate Analysis	OR	95% CI	*P-value*
Head circumference	0.17	0.02–0.64	< 0.05
Abdominal circumference	0.37	0.10–0.78	< 0.05

*AC* abdominal circumference, *CC* chest circumference, *ECMO* extracorporeal membrane oxygenation, *HC* head circumference, *HC/CC* head circumference to chest circumference ratio, *HFOV* high-frequency oscillator ventilation, *iNO* inhaled nitric oxide therapy, *PPHN* persistent pulmonary hypertension

^a^Children with left-sided congenital diaphragmatic hernia only

^b^Age at which pulmonary function test was performed

^c^Measured at birth

^d^Max FiO_2_ denotes maximum oxygen concentration given during ventilator support

^e^Max OI denotes maximum value of oxygen index during hospitalization

^f^Mean OI denotes mean value of oxygen index during hospitalization

^g^Preop Mean OI denotes mean value of oxygen index prior to corrective surgery

After multivariate analysis, smaller head circumference (OR 0.17, *P-value* < 0.05) and abdominal circumference (OR 0.37, *P*-*value* < 0.05) at birth remained significant independent risk factors of abnormal PFT at preschool age (Table 2).

### Chest CT findings of children with repaired CDH

**Fig. 1:**
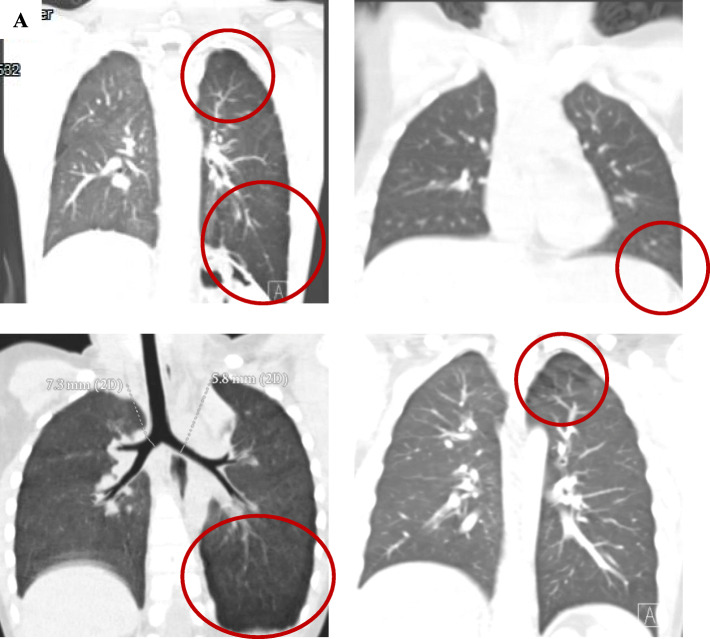
Chest CT scans taken in children with repaired congenital diaphragmatic hernia. Note the multiple emphysematous lesions of the affected lungs in the apical and basal segments (red circles)

Of the children with left CDH (*n* = 28), 10 (36%) were categorized in the TLV < 50 group according to the lung volume measured on CT scans (Supplementary Table 3). The mean LLV/TLV was 47.9 ± 2.5% in the children as a whole; particularly, the TLV ≥ 50 group tended to have a larger volume ratio of the left lung compared with the TLV < 50 group (48.7% vs. 46.6%), albeit the difference was not statistically significant. There were no significant differences in the inner diameters of the left and right main bronchus between the two groups. CT scans in the TLV < 50 group showed multiple pulmonary emphysematous changes in the affected lungs (Fig. 1).

### Comparison of neonatal characteristics in between high and low lung volume groups

To identify the factors associated with the structural growth of lungs in CDH survivors, the neonatal clinical indices were compared between the TLV ≥ 50 group and the TLV < 50 group (Supplementary Table 4). Except for the longer duration of ventilator use in the TLV < 50 group compared with the TLV ≥ 50 group (13.0 vs. 7.1 days; *P-value* < 0.01), the two groups did not show significant differences in any of the perinatal factors, surgical factors, or neonatal factors (all *P-value* > 0.05). Multivariate analysis showed that the risk of having TLV < 50 was significantly lower in children with larger abdominal circumference (OR 0.51, *P-value* < 0.05) and tended to greater in those with excessive amniotic fluid (OR 26.10, *P-value* = 0.06) (Supplementary Table 4).

### Association between chest tomography findings and pulmonary function test results

To analyze the correlation between structural changes in the lung and the functional changes thereof, indices such as TLV, left lung volume, and inner diameters of the main bronchus were compared between the group with normal PFT (*n* = 21) and those with abnormal PFT (*n* = 7). The proportions of children with TLV < 50 in the normal pulmonary function group and the abnormal pulmonary function group were 23.8 and 71.4%, respectively (*P-value* < 0.05). Patients with abnormal pulmonary function associated with smaller lung volume compared to patients with normal pulmonary function (*P-value* < 0.05) (Table 3).

**Table 3:** Association between pulmonary function test and chest tomography^a^

	Normal PFT (*n* = 21)	Abnormal PFT (*n* = 7)	*P-value*
TLV_< 50,_ n (%)	5 (23.8%)	5 (71.4%)	< 0.05
TLV, ml	1244.5 ± 407.9	826.5 ± 133.6	< 0.05
TLV/BSA, ml/m^2^	1515.9 ± 391.3	1017.3 ± 121.8	< 0.05
LLV ratio	48.6 ± 1.8	45.9 ± 3.4	< 0.05
dRMB, mm	8.4 ± 2.0	8.4 ± 1.0	0.9
dLMB, mm	6.3 ± 1.6	6.0 ± 1.0	0.7
dLMB/dRMB	0.8 ± 0.1	0.7 ± 0.1	0.5

*dLMB* diameter of left main bronchus, *dLMB/dRMB* diameter of left main bronchus to diameter of right main bronchus ratio, *dRMB* diameter of right main bronchus, *LLV ratio* left lung volume to total lung volume ratio, *TLV* total lung volume, *TLV/BSA* total lung volume to body surface area ratio *TLV*_*< 50*_ total lung volume below 50%

^a^Children with left-sided congenital diaphragmatic hernia only

## Discussion

In this study, we describe the perinatal-neonatal risk factors in children with repaired CDH with abnormal PFTs and evaluated long-term pulmonary morbidities both in functional and structural aspects. 

Previously, the degrees of impairment are correlated with parameters of disease severity such as liver herniation and patch repair [16]. Accordingly, we found that pulmonary dysfunctions were more likely to develop in children with small head and abdominal circumferences measured at the time of birth. A possible explanation for this finding is that the reduction of head and abdomen circumferences is a consequence of fetal pulmonary growth retardation accompanied by underdevelopment of the lung. Another hypothesis is that large volume of herniated organs in the thoracic cage leads to the restriction of the growth of abdominal organs that have high compliance, while the pressure in the restricted thoracic cage increases and results in the deterioration in pulmonary formation.

Previous studies on the long-term pulmonary functions in children with repaired CDH reported various functional impairments. Children with repaired CDH have lower values of FVC and FEV1 and higher values of residual volume compared with healthy controls, consistent with our observations [11, 16]. Also, a recent study demonstrated that the majority of CDH survivors show fixed pulmonary obstruction with a significant decline of average pulmonary function indices, including FEV_1_ and FVC, over time [9]. These findings emphasize the complex and dynamic change of progressive pulmonary dysfunction in CDH survivors.

With the recent development of imaging modality, multiple studies have investigated the process of lung growth in children with CDH. These MRI studies report catch-up growth of the diseased lung mass between prenatal and postnatal period [12, 13]. Consistent with our results, previous studies also show that children with repaired CDH have parenchymal changes and gas trapping lesions [17, 18]. These findings imply that restrictive pulmonary dysfunction in the early ages of CDH survivors, which was observed in this study and in previous reports, might be related to the gas trapping lesions. In order to elucidate the pathomechanisms associated with pulmonary dysfunction in the early life of CDH survivors, future studies should use multidisciplinary methods to identify the relationship between RV and/or FRC and pulmonary over-distention observed in chest CT.

The pathogenesis of the mechanism in CDH and the compensatory growth of the disease-involved lung is not well-known. An analysis of lung tissues of aborted fetuses with CDH showed that the gross structural and histological changes in the affected lungs occur between 27 and 30 weeks of gestation age [4], which is the period of active development of the bronchioles and alveolar cysts during the transition from the tubular to the follicular phase. Patients who have undergone pulmonary hypoplasia at this time will continue to go through underdevelopment of the alveolus in the future. The lungs continuously grow and maturate even after birth, mainly by the increase in air conduction [19–21]. Throughout childhood, the diameter of the airway including the bronchus grows approximately three times more than at birth. The growth continues until 5 years of age, and occurs more actively in the peripheral airway than in the central airway. Alveoli also grow quantitatively and qualitatively until 2 to 3 years of age in proportion to the increases in body weight [22, 23]. Therefore, children with CDH may have smaller degrees of obstruction due to the relatively active peripheral airway development, while restrictive pulmonary impairment stemming from abnormal fetal lung alveolarization process may contribute to the occurrence of restrictive pulmonary impairment due to impaired alveolarization. In fact, children with repaired CDH showing restrictive pulmonary function also showed compensatory pulmonary emphysematous changes in the affected lung.

The limitations of this study are as follows. First, whereas this study focused on function and structural changes in the lungs, pulmonary growth is closely related to the development of cardiovascular growth. As such, subsequent studies should also evaluate pulmonary artery growth, pulmonary hypertension, and cardiovascular anomalies. Second, the results of the pulmonary volume measured by pulmonary CT were not compared with that of the healthy control group. As the normal value of lung volume according to age is yet to be elucidated, we predicted the changes in the left lung volume through the left lung volume ratio measured in the supine position.

## Conclusion

In this study, we evaluated the relationship between structural findings obtained in CT scans and lung function measured by pulmonary function tests in preschool children with repaired left CDH and identified the associated neonatal-perinatal risk factors. We found that a quarter of long-term surviving children with repaired CDH showed deteriorated findings in pulmonary function test around the age of 6, most of whom had restrictive patterns. Children with smaller abdominal and head circumferences were more likely to have pulmonary impairment at preschool age. Furthermore, we found that the risk of pulmonary dysfunction decreased with greater lung volume after growth. Collectively, our findings suggest that the deterioration in structural pulmonary growth leads to pulmonary dysfunction.

## Supplementary Information

**Additional file 1: Supplementary Table 1**. Neonatal characteristics of preschool children with repaired congenital diaphragmatic hernia. **Supplementary Table 2**. Surgical findings and respiratory support of preschool children with repaired congenital diaphragmatic hernia. **Supplementary Table 3**. Computed tomography findings in children with repaired congenital diaphragmatic hernia. **Supplementary Table 4**. Perinatal, surgical, and neonatal variables in children with high (≥ 50) and low (< 50) total lung volume.

## Data Availability

The data that support the findings of this study are available from the corresponding author on request.

## Abbreviations

CDH: Congenital diaphragmatic hernia
CT: Chest computed tomography
FEV1: Forced expiratory volume in 1 s
FEV1/FVC: FEV1/FVC ratio
FVC: Forced vital capacity
PFT: Pulmonary function tests
TLV: Total lung volume

## References

[1] Daodu O, Brindle ME (2017). Predicting outcomes in congenital diaphragmatic hernia. Semin Pediatr Surg.

[2] Wright JC, Budd JL, Field DJ (2011). Epidemiology and outcome of congenital diaphragmatic hernia: a 9-year experience. Paediatr Perinat Epidemiol.

[3] Kitagawa M, Hislop A, Boyden EA (1971). Lung hypoplasia in congenital diaphragmatic hernia. A quantitative study of airway, artery, and alveolar development. Br J Surg.

[4] Bargy F, Beaudoin S, Barbet P (2006). Fetal lung growth in congenital diaphragmatic hernia. Fetal Diagn Ther.

[5] Sakai H, Tamura M, Hosokawa Y (1987). Effect of surgical repair on respiratory mechanics in congenital diaphragmatic hernia. J Pediatr.

[6] Lund DP, Mitchell J, Kharasch V (1994). Congenital diaphragmatic hernia: the hidden morbidity. J Pediatr Surg.

[7] Downard CD, Jaksic T, Garza JJ (2003). Analysis of an improved survival rate for congenital diaphragmatic hernia. J Pediatr Surg.

[8] Antonoff MB, Hustead VA, Groth SS (2011). Protocolized management of infants with congenital diaphragmatic hernia: effect on survival. J Pediatr Surg.

[9] Dao DT, Hayden LP, Buchmiller TL (2020). Longitudinal analysis of pulmonary function in survivors of congenital diaphragmatic hernia. J Pediatr.

[10] Dao DT, Kamran A, Wilson JM (2020). Longitudinal analysis of ventilation perfusion mismatch in congenital diaphragmatic hernia survivors. J Pediatr.

[11] Panitch HB, Weiner DJ, Feng R (2015). Lung function over the first 3 years of life in children with congenital diaphragmatic hernia. Pediatr Pulmonol.

[12] Adaikalam SA, Higano NS, Tkach JA (2019). Neonatal lung growth in congenital diaphragmatic hernia: evaluation of lung density and mass by pulmonary MRI. Pediatr Res.

[13] Schopper MA, Walkup LL, Tkach JA (2017). Evaluation of neonatal lung volume growth by pulmonary magnetic resonance imaging in patients with congenital diaphragmatic hernia. J Pediatr.

[14] Quanjer PH, Stanojevic S, Cole TJ (2012). Multi-ethnic reference values for spirometry for the 3-95-yr age range: the global lung function 2012 equations. Eur Respir J.

[15] Gollogly S, Smith JT, White SK (2004). The volume of lung parenchyma as a function of age: a review of 1050 normal CT scans of the chest with three-dimensional volumetric reconstruction of the pulmonary system. Spine (Phila Pa 1976).

[16] Wigen RB, Duan W, Moraes TJ (2019). Predictors of long-term pulmonary morbidity in children with congenital diaphragmatic hernia. Eur J Pediatr Surg.

[17] Tan JK, Banton G, Minutillo C (2019). Long-term medical and psychosocial outcomes in congenital diaphragmatic hernia survivors. Arch Dis Child.

[18] Wright T, Filbrun A, Bryner B (2014). Predictors of early lung function in patients with congenital diaphragmatic hernia. J Pediatr Surg.

[19] Cotten CM (2017). Pulmonary hypoplasia. Semin Fetal Neonatal Med.

[20] Lundin A, Driscoll B (2013). Lung cancer stem cells: progress and prospects. Cancer Lett.

[21] Schittny JC (2017). Development of the lung. Cell Tissue Res.

[22] Herring MJ, Putney LF, Wyatt G (2014). Growth of alveoli during postnatal development in humans based on stereological estimation. Am J Phys Lung Cell Mol Phys.

[23] Narayanan M, Owers-Bradley J, Beardsmore CS (2012). Alveolarization continues during childhood and adolescence: new evidence from helium-3 magnetic resonance. Am J Respir Crit Care Med.

