# Supplementary material for: Functional and structural evaluation in the lungs of children with repaired congenital diaphragmatic hernia
Source: BMC Pediatr. 2021 Mar 11;21:120. doi: 10.1186/s12887-021-02586-3 (PMC7947149; doi:10.1186/s12887-021-02586-3)
Supplement: Supplementary file 1 — Additional file 1: Supplementary Table 1. Neonatal characteristics of preschool children with repaired congenital diaphragmatic hernia. Supplementary Table 2. Surgical findings and respiratory support of preschool children with repaired congenital diaphragmatic hernia. Supplementary Table 3. Computed tomography findings in children with repaired congenital diaphragmatic hernia. Supplementary Table 4. Perinatal, surgical, and neonatal variables in children with high (≥ 50) and low (< 50) total lung volume. [file 12887_2021_2586_MOESM1_ESM.docx]

**Functional and Structural Evaluation in the Lungs of Children with Repaired Congenital Diaphragmatic Hernia**

June-Young Koh, M.D.^1,2^, Euiseok Jung, M.D. ^1^, Hyun Woo Goo, M.D.Ph.D.^3^, Seong-Chul Kim, M.D.Ph.D.^4^, Dae Yeon Kim, M.D.Ph.D.^4^, Jung-Man Namgoong, M.D.Ph.D.^4^, Byong Sop Lee, M.D.Ph.D.^1^, Ki-Soo Kim, M.D.Ph.D.^1^, and Ellen Ai-Rhan Kim, M.D.Ph.D. ^1^*

^1^ Departments of Pediatrics, Asan Medical Center, University of Ulsan College of Medicine, Seoul, Republic of Korea

^2^ Graduate School of Medical Science and Engineering, Korea Advanced Institute of Science and Technology (KAIST), Daejeon, Republic of Korea

^3^ Department of Radiology and Research Institute of Radiology, Asan Medical Center, University of Ulsan College of Medicine, Seoul, Republic of Korea

^4^ Departments of Pediatric Surgery, Asan Medical Center, University of Ulsan College of Medicine, Seoul, Republic of Korea

* Corresponding author:

Ellen Ai-Rhan Kim, M.D.Ph.D.

Division of Neonatology, Department of Pediatrics, Asan Medical Center Children’s Hospital, University of Ulsan College of Medicine

88, Olympic-ro 43-gil, Songpa-gu, Seoul 05505, Republic of Korea

Tel: +82-2-3010-3382, Fax: +82-2-3010-6978, E-mail: [arkim@amc.seoul.kr](mailto:arkim@amc.seoul.kr)

Supplementary Table 1. Neonatal characteristics of preschool children with repaired congenital diaphragmatic hernia

|  | Total (n = 30) |
| --- | --- |
| Male, n (%) | 17 (57%) |
| Birth weight, g | 2964.8 ± 315.2 |
| Gestational age, weeks | 38.4 ± 1.0 |
| 1 min APGAR score | 5.9 ± 1.5 |
| 5 min APGAR score | 7.8 ± 1.1 |
| HC^*^, cm | 34.6 ± 1.2 |
| CC^*^, cm | 31.8 ± 1.6 |
| AC^*^, cm | 28.7 ± 2.2 |
| HC/CC^*^ | 1.1 ± 0.1 |
| Polyhydramnios, n (%) | 4 (13%) |
| Associated anomaly^†^, n (%) | 1 (3%) |

*AC*, abdominal circumference; *CC*, chest circumference; *HC*, head circumference; *HC/CC*, head circumference to chest circumference ratio.

^*^Measured at birth.

^†^Associated anomaly includes 1 case of omphalocele.

Supplementary Table 2. Surgical findings and respiratory support of preschool children with repaired congenital diaphragmatic hernia

|  | Total (n = 30) |
| --- | --- |
| Surgical findings |  |
| Day of surgery after birth | 4.1 ± 1.8 |
| Left sided defect, n (%) | 28 (93%) |
| Defect size, cm^2^ | 11.8 ± 9.2 |
| Presence of hernia sac, n (%) | 16 (53%) |
| Herniated organs, n | 2.9 ± 1.0 |
| Stomach, n (%) | 13 (43%) |
| Small bowel, n (%) | 29 (97%) |
| Colon, n (%) | 23 (77%) |
| Liver, n (%) | 5 (17%) |
| Spleen, n (%) | 19 (63%) |
| Kidney, n (%) | 1 (3%) |
| Respiratory support |  |
| Cardiorespiratory support, n (%) | 30 (100%) |
| High frequency oscillator ventilation, n (%) | 11 (37%) |
| Inhaled nitric oxide therapy, n (%) | 2 (7%) |
| Extracorporeal Membrane Oxygenation, n (%) | 1 (3%) |
| Max FiO_2_^*^ | 0.5 ± 0.2 |
| Max OI^†^ | 10.3 ± 13.8 |
| Mean OI^‡^ | 3.8 ± 2.7 |
| Duration of ventilation, days | 8.9 ± 6.2 |
| Persistent pulmonary hypertension, n (%) | 14 (47%) |
| Pneumothorax, n (%) | 1 (3%) |

^*^Max FiO_2_ denotes maximum oxygen concentration given during ventilator support.

^†^Max OI denotes maximum value of oxygen index during hospitalization.

^‡^Mean OI denotes mean value of oxygen index during hospitalization.

Supplementary Table 3. Computed tomography findings in children with repaired congenital diaphragmatic hernia^*^

|  | Total (n = 28) | TLV_≥50_ (n = 18) | TLV_<50_ (n = 10) | *P-value* |
| --- | --- | --- | --- | --- |
| CT age^†^, years | 6.2 ± 1.0 | - | - | - |
| TLV, mL | 1140.0 ± 401.5 | 1339.1 ± 363.9 | 781.7 ± 107.3 | <.0001 |
| TLV/BSA, mL/m^2^ | 1391.3 ± 406.3 | 1619.4 ± 309.6 | 980.7 ± 150.6 | <.0001 |
| LLV ratio | 47.9 ± 2.5 | 48.7 ± 1.9 | 46.6 ± 3.1 | .08 |
| dRMB, mm | 8.4 ± 1.8 | 8.4 ± 2.2 | 8.3 ± 0.7 | .9 |
| dLMB, mm | 6.2 ± 1.4 | 6.0 ± 1.7 | 6.5 ± 0.9 | .3 |
| dLMB/dRMB | 0.8 ± 0.1 | 0.7 ± 0.1 | 0.8 ± 0.1 | .1 |

dLMB, diameter of left main bronchus; *dLMB/dRMB*, diameter of left main bronchus to diameter of right main bronchus ratio; *dRMB*, diameter of right main bronchus; *LLV ratio*, left lung volume to total lung volume ratio; *TLV*, total lung volume; *TLV/BSA*, total lung volume to body surface area ratio.

^*^Children with left-sided congenital diaphragmatic hernia only.

^†^Age at which computed tomography was performed.

Supplementary Table 4. Perinatal, surgical, and neonatal variables in children with high (≥ 50) and low (< 50) total lung volume ^*^

|  | | TLV_≥50_ (n = 18) | TLV_<50_ (n = 10) | | *P-value* | |  |
| --- | --- | --- | --- | --- | --- | --- | --- |
| CT age^†^, years | | 6.3 ± 1.1 | 6.3 ± 0.9 | | >.99 | |  |
| Perinatal factors | |  |  | |  | |  |
| Male, n (%) | | 11 (61%) | 4 (40%) | | 0.2 | |  |
| Birth weight, g | | 3007.6 ± 320.1 | 2898.6 ± 325.1 | | 0.4 | |  |
| Gestational age, weeks | | 38.4 ± 1.0 | 38.4 ± 1.2 | | 0.9 | |  |
| 1 min APAGAR score | | 6.1 ± 1.7 | 5.9 ± 1.2 | | 0.8 | |  |
| 5 min APAGAR score | | 7.9 ± 1.2 | 7.6 ± 1.1 | | 0.5 | |  |
| HC^‡^, cm | | 34.6 ± 1.1 | 34.5 ± 1.4 | | 0.8 | |  |
| CC^‡^, cm | | 32.0 ± 1.4 | 31.5 ± 2.0 | | 0.4 | |  |
| AC^‡^, cm | | 29.3 ± 1.7 | 27.8 ± 2.8 | | 0.1 | |  |
| HC/CC^‡^ | | 1.1 ± 0.04 | 1.1 ± 0.08 | | 0.4 | |  |
| Polyhydramnios, n (%) | | 1 (6%) | 3 (30%) | | 0.2 | |  |
| Surgical findings | |  |  | |  | |  |
| Herniated organs, n | | 3 ± 1.0 | 3 ± 0.9 | | >.99 | |  |
| Stomach, n (%) | | 8 (44%) | 5 (50%) | | 0.6 | |  |
| Small bowel, n (%) | | 17 (94%) | 9 (90%) | | 0.4 | |  |
| Colon, n (%) | | 13 (72%) | 8 (80%) | | 0.6 | |  |
| Liver, n (%) | | 2 (11%) | 2 (20%) | | 0.5 | |  |
| Spleen, n (%) | | 10 (56%) | 8 (80%) | | 0.2 | |  |
| Kidney, n (%) | | 1 (6%) | 0 (0%) | | 0.6 | |  |
| Defect size, cm^2^ | | 11.7 ± 6.1 | 12.5 ± 13.7 | | 0.9 | |  |
| Neonatal factors | |  |  | |  | |  |
| Days to surgery | | 3.9 ± 1.8 | 4.3 ± 1.8 | | 0.6 | |  |
| HFOV, n (%) | | 6 (33%) | 5 (50%) | | 0.3 | |  |
| iNO, n (%) | | 0 (0%) | 2 (20%) | | 0.1 | |  |
| ECMO, n (%) | | 0 (0%) | 1 (10%) | | 0.4 | |  |
| Max FiO_2_^§^ | | 0.5 ± 0.3 | 0.5 ± 0.3 | | 0.8 | |  |
| Max OI^?^ | | 10.0 ± 15.4 | 11.7 ± 11.6 | | 0.7 | |  |
| Mean OI^¶^ | | 3.8 ± 2.1 | 4.2 ± 3.7 | | 0.7 | |  |
| Preop Mean OI^**^ | | 5.7 ± 5.0 | 6.0 ± 6.5 | | 0.9 | |  |
| Duration of ventilator, days | | 7.1 ± 3.4 | 13.0 ± 8.2 | | < 0.05 | |  |
| Hospital days | | 21.7 ± 9.9 | 28.3 ± 15.5 | | 0.2 | |  |
| PPHN, n (%) | | 7 (39%) | 6 (60%) | | 0.2 | |  |
| Pneumothorax, n (%) | | 1 (6%) | 0 (0%) | | 0.6 | |  |
| Multivariate Analysis |  | | |  | |  | |
|  | | OR | 95% CI | | *P-value* | |  |
| Abdominal circumference | | 0.51 | 0.23–0.86 | | < 0.05 | |  |
| Polyhydramnios | | 26.10 | 1.47–1569.91 | | 0.06 | |  |

*AC*, abdominal circumference; *CC*, chest circumference; *ECMO*, extracorporeal membrane oxygenation; *HC*, head circumference; *HC/CC*, head circumference to chest circumference ratio; *HFOV*, high-frequency oscillator ventilation; *iNO*, inhaled nitric oxide therapy; *PPHN*, persistent pulmonary hypertension; *TLV_≥50_*, total lung volume above 50%; *TLV_<50_*, total lung volume below 50%.

^*^ Children with left-sided congenital diaphragmatic hernia only.

^†^Age at which computed tomography performed.

^‡^Measured at birth.

^§^Max FiO_2_ denotes maximum oxygen concentration given during ventilation care.

^?^Max OI denotes maximum value of oxygen index during hospitalization.

^¶^Mean OI denotes average value of oxygen index during hospitalization.

^**^Pre op OI denotes average value of oxygen index prior to surgery.
